# Deloading Practices in Strength and Physique Sports: A Cross-sectional Survey

**DOI:** 10.1186/s40798-024-00691-y

**Published:** 2024-03-18

**Authors:** David Rogerson, David Nolan, Patroklos Androulakis Korakakis, Velu Immonen, Milo Wolf, Lee Bell

**Affiliations:** 1 Academy of Sport and Physical Activity, Sheffield Hallam University, Sheffield, S10 2BP UK; 2 School of Health & Human Performance, Dublin City University, Dublin, Ireland; 3 Department of Exercise Science and Recreation, Applied Muscle Development Laboratory, CUNY Lehman College, Bronx, NY USA; 4 Department of Sports and Exercise, Haaga-Helia University of Applied Sciences, Vierumäki, 19120 Finland; 5 Centre for Health, Exercise and Sport Science, Solent University, E Park Terrace, Southampton, SO14 0YN UK

**Keywords:** Deloading, Strength Training, Strength Sports, Bodybuilding

## Abstract

**Background:**

This study explored the deloading practices of competitive strength and physique athletes. A 55-item anonymised web-based survey was distributed to a convenience-based, cross-sectional sample of competitive strength and physique athletes (*n =* 246; males = 181 [73.6%], females = 65 [26.4%]; age = 29.5 ± 8.6 years) who had 8.2 ± 6.2 years of resistance training and 3.8 ± 3.1 years of competition experience.

**Results:**

All athletes deloaded within training with energy and fatigue management being the main reasons to do so. The typical duration of a deload was 6.4 ± 1.7 days, integrated into the training programme every 5.6 ± 2.3 weeks. Deloading was undertaken using a proactive, pre-planned strategy (or in combination with an autoregulated approach) and undertaken when performance stalled or during periods of increased muscle soreness or joint aches. Athletes reported that training volume would decrease (through a reduction in both repetitions per set and sets per week), but training frequency would remain unchanged during deloads. Additionally, athletes reported that training intensity (load lifted) would decrease, and effort would be reduced (facilitated through an increase in repetitions in reserve). Athletes would generally maintain the same exercise selection during deloading. For athletes that supplemented deloading with additional recovery modalities (*n =* 118; 48%), the most reported strategies were massage, static stretching and foam rolling.

**Conclusion:**

Results from this research might assist strength and physique athletes and coaches to plan their deloading. Future research should empirically investigate the findings from this study to further evaluate the potential utility of deloading in strength and physique sports.

## Background

Athletes and recreational trainees participate in resistance exercise to enhance their athletic potential and to improve their musculature and appearance [1, 2]. Whilst strength training is an important part of an athlete’s physical preparation, strength sports such as Powerlifting, Weightlifting and Strongman/woman competitions are globalised sports where athletes compete to determine who is the strongest within the parameters of their lifts and/or events [3–5]. Similarly, within physique sports such as bodybuilding, individuals participate in competitions to see who is the most symmetrical, muscular, and conditioned [6], utilising resistance training to build and refine their physiques [7]. Such activities can be loosely categorised as strength and physique sports, and each share the need for participants to engage with resistance training to be competitive. The development of strength requires the manipulation of training variables such as volume, frequency, and intensity to elicit adaptive responses within the neuromuscular system that enhance volitional force production, such as increased intramuscular and intermuscular coordination and the disinhibition of inhibitory mechanisms [8–10]. Similarly, resistance training provokes adaptive responses within skeletal muscle that increase cross-sectional area incrementally as muscle protein accretion accumulates to observable levels, typically evident after 6 or more weeks of training [11]. This is despite hypertrophic responses being immediate upon stimulation through resistance exercise, however [12, 13]. In all instances, the continued development of such neural and morphological adaptations requires exercise to be progressive, which over time, might require that the training becomes more challenging and sophisticated as the athlete becomes better conditioned to its demands [14].

The strategic and phasic planning of training around key competitive activities is the hallmark of periodisation, where training is organised in such a way as to elicit adaptive responses in either a sequential or parallel manner, oftentimes within a cyclical format [15]. Typically, with periodised training, these cycles are organised over longer (macro), medium (meso) and shorter (micro) timeframes and with clear purpose in relation to task and athlete-specific needs and competitive schedules [15]. For training to be stimulatory, it needs to be of sufficient magnitude to elicit an adaptive response [14]. However, fatigue is also an important byproduct of strenuous training [16], and a well thought out, periodised programme will make use of unloading phases and cycles to dissipate fatigue and allow for training to continue without maladaptation, or for the cumulative effects of consistent training to be realised within competition [17]. It should be noted, however, that evidence for accumulated fatigue within resistance training is surprisingly indistinct at this time, and that accumulated ‘fatigue’ might in fact be muscle damage accrued from hard training, which shares similar physiological characteristics of delayed appearance and reduced force production [16]. Nonetheless, without sufficient recuperation, performance can become affected by non-functional overreaching [18], and recovery periods are required to reduce the negative consequences of consistent, progressive training. Periods of reduced training are often referred to as ‘tapering’ or ‘peaking’ strategies when preparing for competition, or ‘unloading’ or ‘deloading’ cycles or phases within day-to-day training [19]. Indeed, a well-designed training programme will often make use of both to facilitate day-to-day training and recovery as well as competition readiness.

Tapering is a short period of reduced training stress strategically completed in the days/weeks leading up to competition, to facilitate a ‘peak’ for their specific event by optimising their readiness through the reduction of fatigue and optimisation of performance [19]. The practice is well established, with up to 87–99% of strength athletes incorporating tapering according to research elsewhere [20, 21]). For strength sports such as powerlifting, strongman and weightlifting, a reduction in training volume whilst maintaining or reducing intensity appears to be the most common approach [20–23]. For these athletes, tapering is often undertaken for a period of ~ 7 days (and, often following a peak in training volume in the weeks prior), with training cessation commencing 4 ± 2 days prior to competing [20–23]. Tapering practices appear to differ in physique sports such as bodybuilding however, where athletes appear to maintain resistance training whilst manipulating dietary strategies (e.g., energy balance, macronutrient intake, hydration, and sodium) and physical activity levels (e.g., cardiovascular exercise, step count) to optimise their on-stage appearance in the days leading up to competition [24, 25]. The lack of tapering in physique vs. strength sports most likely reflects the different objectives of the two activities: Physique sports celebrate an aesthetic ideal and use resistance training as a tool to build the physique prior to competing [24, 25]; strength sports prioritise physical performance and use resistance training as a tool to build the physical abilities that are then realised in competition [24]. The strategic reduction in training stress to ‘peak’ towards a discrete physical performance is perhaps only applicable to the latter therefore [24].

Despite almost universal usage within practice and growing evidence exploring tapering, research exploring deloading within strength and physique sports is somewhat sparse, and it appears that previous discussion has even used the terms deloading and tapering interchangeably [26]. This suggests that terminological confusion might also coexist alongside a lack of data within the literature. Despite this, recent work by our team explored coaches’ perceptions and experiences of deloading within strength and physique sports through qualitative methods, highlighting that coaches strategically utilised deloading to manage fatigue and facilitate longer-term progression [27]. Interestingly, our results also revealed that practises varied considerably, with periods of reduced volume, intensity of effort, and exercise mode and configuration programmed every 4–6 weeks for a duration of 5–7 days in an individualised manner, adapted to the needs and context of athletes. Indeed, data demonstrates that individuals can respond differently to resistance training, and that training might therefore need to be individualised for it to be optimal [28]. It stands to reason therefore that approaches taken to strategically deload from training might also require some degree of personalisation too. Whilst we explored coaches’ practices previously, we did not investigate the practices of competitive strength and physique athletes per se, who might adopt nuanced and unique approaches and have different experiences and methods than coaches. The investigation of the deloading approaches taken by individual athletes who participate in resistance training sports might be warranted, therefore, to gain a deeper understanding of its usage within practice.

Given that deloading is an under researched but almost ubiquitous aspect of strength and physique athlete training, it is important to understand current practices and add to the existing – but sparse – evidence in this area. Therefore, the purposes of this study were to (1), investigate the deloading practices of strength and physique athletes; and (2), develop an understanding of the rationale used by such athletes when implementing deloading into their training. It was anticipated that the outcomes of this work would be of interest to researchers, athletes, and coaches alike, who all might need to programme deloading within training programmes and interventions.

## Methods

### Survey Development

Following institutional ethical approval (ER38311849), an open, anonymous, cross-sectional survey was developed using Typeform (Typeform SL, Barcelona, Spain), a secure online software service that specialises in online surveys. The survey was created collaboratively by the research team. Questions were developed pragmatically and guided by previous research [27]. To enhance validity and to ensure all relevant questions were captured, all members of the research team evaluated and provided feedback on the quality, accuracy, and scope of the survey in relation to the aims of the study. Each member of the research team had either coached or participated in strength and physique sports and programmed strength training alongside undertaking research in the field and were well placed to review, critique, and develop the questions in this way. The survey was then piloted with participants who shared the same characteristics as the inclusion criteria, to establish face validity and inform further revisions, if necessary [29]. Based on the resulting feedback, the survey was then refined for readability and clarity. Before making the survey available for completion by participants, all members of the research team then independently tested the survey interface to ensure appropriate useability. This process of development, pre-testing, piloting, and refinement helped to quality assure the survey prior to its administration and reflects recommended processes as suggested elsewhere [30].

The final version of the survey was available online (https://deload.survey) between November 2021 and March 2022. The final survey consisted of 55 questions and was presented as multiple-choice or open-format responses based on the type of question. The survey contained adaptive questioning with several questions conditionally displayed based on responses to previous items. More specifically, sport-specific questions were displayed based on selected sport (i.e., strength or physique sport), and clarifying questions were displayed to participants that indicated the use of nutritional changes, deload enjoyment, and training breaks. To reduce the risk of multiple entries from the same participant, individuals were assigned a unique user identification number based on their internet protocol (IP) address and given an opportunity to review and change their answers throughout the completion of the survey. No duplicate responses were detected.

### Sample Selection and Recruitment

A voluntary convenience sample of strength and physique athletes was recruited through social media and emails to industry experts/gatekeepers involved in relevant sports across the globe. Participants were eligible if they currently used deloads as part of their overall training programme and competed in strength or physique sports. A recruitment poster was designed to outline the purpose of the research, eligibility criteria and a direct link to the website and survey (via QR code).

The sampling criteria specified that participants were ≥ 18 years of age and have competed in either a strength or physique sport and currently use deloading as part of their overall training programme. No restriction was placed on the level of competition (i.e., club to international level athletes) or federation. For the purposes of this research, eligible sports were categorised as strength sports (weightlifting, powerlifting and strongman) and physique sports (bodybuilding in all forms). The choice of sports was determined using previous research on deloading practices in strength and physique sports [27]. To provide sufficient clarity regarding deloading but to avoid acquiesce bias, the term “deloading” was broadly defined as involvement in any training that involved a planned reduction in training stress such as “light weeks” or “recovery weeks”.

### Statistical Analysis

Statistical analysis of the anonymised data was conducted using the Tidyverse package in R statistical software (version 4.0.5). Mean and *SD* demographic data were calculated for the whole participant group, as well as subgroups of sex, age, resistance exercise training experience, and competition experience/level. The range was calculated for discrete variables (resistance training experience, competing experience, how often you use deloads and how long do you deload for). Deloading characteristics were categorised for the whole group and according to the participants’ sport.

## Results

### Demographic and Training Characteristics

Thirty-two participants were excluded due to not meeting the inclusion criteria (18 had not used deloads and 14 had previously used deloads in their training, but not currently). The responses of 246 athletes were included in the analysis. The mean and *SD* age of participants was 29.5 ± 8.6 years. One-hundred and eighty-one (73.6%) were male and 65 (26.4%) were female. One hundred and fifty-six (63.4%) athletes were powerlifters; 9 (3.7%) were weightlifters; 9 (3.7%) were strongman athletes; 45 (18.3%) were physique athletes and 27 (11.0%) were classified as mixed athletes (involved in > 1 of the sports mentioned above). On average, athletes had 8.2 ± 6.2 years of resistance exercise training experience and had competed in their respective strength or physique sport for 3.8 ± 3.2 years. Competition level included 47 (19.1%) athletes who competed at an international level, 84 (34.1%) at a national level, 36 (14.6%) at a regional level, and 79 (32.1%) who competed at a state/local level. Most athletes reported having a coach (54.1%), with the remainder being self-coached (45.9%). A summary of demographic and training characteristics for all athletes is presented in Table 1.

**Table 1:** Demographics and training characteristics

	All athletes (*n* = 246)	Powerlifting (*n* = 156; 63.4%)	Weightlifting (*n* = 9; 3.7%)	Strongman/woman (*n* = 9; 3.7%)	Physique (*n* = 45; 18.3%)	Mixed* (*n* = 27; 11.0%)
***Demographics***						
Sex (M = male; F = female)	M = 181 (73.6%) F = 65 (26.4%)	M = 110 (70.5%) F = 46 (29.5%)	M = 8 (88.9%) F = 1 (11.1%)	M = 8 (88.9%) F = 1 (11.1%)	M = 31 (68.9%) F = 14 (31.1%)	M = 24 (88.9%) F = 3 (11.1%)
Age (Years) (Mean ± *SD*) Range Min Max	29.5 ± 8.6 45 18 63	29.1 ± 9.3 45 18 63	26.9 ± 4.4 13 21 34	28.4 ± 4.1 13 24 35	30.0 ± 7.0 29 18 47	32.5 ± 8.9 37 18 55
***Coached***						
Coached	113 (45.9%)	91 (58.3%)	4 (44.4%)	4 (44.4%)	23 (51.1%)	11 (40.7%)
Self-coached	133 (54.1%)	65 (41.7%)	5 (55.6%)	5 (55.6%)	22 (48.9%)	16 (59.3%)
***Training characteristics***						
Resistance training experience (Years) (Mean ± *SD*) Range Min Max	8.2 ± 6.2 44 1 45	7.1 ± 5.3 44 1 45	9.8 ± 5.1 18 3 20	8.8 ± 4.4 15 4 19	9.3 ± 6.3 30 1 31	12.3 ± 9.2 41 2 43
Competition experience (Years) (Mean ± *SD*) Range Min Max	3.8 ± 3.2 35 1 21	3.5 ± 2.5 14 1 15	5.2 ± 3.1 9 2 11	3.3 ± 2.3 7 1 8	4.0 ± 4.2 20 1 21	4.9 ± 4.6 36 1 37
***Competition level***						
International (*n*)	47 (19.1%)	28 (17.9%)	-	4 (44.4%)	9 (20.0%)	6 (22.2%)
National (*n*)	84 (34.1%)	50 (32.1%)	5 (55.6%)	2 (22.3%)	17 (37.8%)	10 (37.0%)
Regional (*n*)	36 (14.6%)	27 (17.3%)	1 (11.1%)	-	5 (11.1%)	3 (11.1%)
State / local (*n*)	79 (32.1%)	51 (32.7%)	3 (33.3%)	3 (33.3%)	14 (31.1%)	8 (29.6%)

*Mixed athletes participate in > 1 of the sports included in this study

### Reasons for Implementing Deloading

All included athletes (*n* = 246) indicated they currently use deloading as part of their overall training programme. A summary of responses is presented in Table 2. The most common reasons for implementing deloading reported by athletes were to decrease fatigue (92.3%), prepare for a change in training (e.g., between training blocks) (64.6%), and to improve performance (59.8%). Most athletes (65.0%) stated that they felt they could progress in their training without deloading.

**Table 2:** Reasons for deloading

	Responses (*n* = 246)
***“Do you think you could progress without deloads?”***	
Yes	160 (65.0%)
No	86 (35.0%)
***“Why do you use deloads?”***	
Decrease fatigue	227 (92.3%)
Prepare for a change in training (e.g., between training blocks)	159 (64.6%)
Improve performance	147 (59.8%)
To prepare for a competition	126 (51.2%)
Psychological reasons	119 (48.4%)
Injury prevention	119 (48.4%)
Injury management	80 (32.5%)
Maintain performance and/or muscle mass	52 (21.1%)
To preserve energy for other things (e.g., non-training life stressors)	46 (18.7%)
Sleep disruption	25 (10.2%)
Increase muscle mass	19 (7.7%)

### Duration and Frequency of Deloading

Athletes (*n* = 246) stated that deloading would be undertaken every 5.6 ± 2.3 weeks (Range = 11, Min = 1, Max = 12 weeks). The typical deload for all respondents was 6.4 ± 1.7 days in duration (Range = 13, Min = 1, Max = 14 days). A summary of the duration and frequency of the deload for all groups can be found in Table 3.

**Table 3:** Duration and frequency of deloading

	All athletes (*n* = 246)	Powerlifting (*n* = 156; 63.4%)	Weightlifting (*n* = 9; 3.7%)	Strongman/woman (*n* = 9; 3.7%)	Physique (*n* = 45; 18.3%)	Mixed (*n* = 27; 11.0%)
***“How often do you take deloads?”*** (Weeks)						
(Mean ± *SD*) Range Min Max	5.6 ± 2.3 11 1 12	5.5 ± 2.2 11 1 12	4.8 ± 1.1 6 2 8	6.7 ± 3.4 11 1 12	5.8 ± 2. 11 1 12	5.7 ± 2.5 11 1 12
***“How long do you usually deload for?”*** (Days)						
(Mean ± *SD*) Range Min Max	6.4 ± 1.7 13 1 14	6.5 ± 1.6 13 1 14	8.5 ± 1.6 7 3 10	6.3 ± 1.0 2 7 5	6.3 ± 2.1 13 1 14	6.2 ± 1.4 6 1 7

### When Deloading is Implemented

Athletes (*n* = 246) provided information related to when they chose to deload. A summary of responses is presented in Table 4. Many participants (47.2%) planned their deloads proactively, using a pre-planned approach compared to an autoregulated approach (13.4%). Some athletes used a combination of reactive and proactive strategies (39.4%). The main reasons for implementing deloading were when it said so on the programme (65.4%), when feeling beat up (muscle soreness, joint aches, or pain) (62.6%) and when performance stalled or decreased (54.1%). “Other” (0.8%) reasons for deloading were related to increased fatigue.

**Table 4:** When deloading is implemented

	Responses (*n* = 246)
**“When do you deload?”**	
Pre-planned	116 (47.2%)
Both Pre-planned and Reactively (autoregulation)	97 (39.4%)
Reactively (autoregulation) only	33 (13.4%)
***“When do you use deloads?”***	
When it says so on the programme	161 (65.4%)
When feeling beat up (muscle soreness, joint aches, or pain)	154 (62.6%)
When performance stalls or decreases	113 (54.1%)
When previous injuries start acting up	77 (31.3%)
When dealing with high levels of external stress (e.g., work, family, relationships, etc.)	75 (30.5%)
When I’m on vacation, out of town etc.	60 (24.4%)
When I don’t feel like training	36 (14.6%)
Other	2 (0.8%)

### Changes in Training Frequency and Volume During Deloading

Athletes (*n* = 246) reported how they would adapt training frequency and volume during a period of deloading. A summary of responses is presented in Table 5. Most participants reported that the number of weekly training sessions would not change (63.0%) or would decrease (32.9%) during deloading. Similarly, the frequency of competition lifts or main multi-joint exercises would also remain unchanged (61.0%) or decrease (32.9%). “Other” (1.2%) reasons suggested that the frequency of competition lifts might increase or decrease depending on the specific circumstances of the athlete. Most athletes stated that the number of weekly sets would decrease (78.9%) or remain unchanged (17.9%), and that the number of repetitions performed within each set would also likely decrease (52.8%) or remain the same (30.9%).

**Table 5:** Changes in training frequency and volume during deloading

	Responses (*n* = 246)
***Changes in weekly training sessions (training frequency)***	
No change	155 (63.0%)
Decrease	81 (32.9%)
Increase	10 (4.1%)
Other	0 (0.0%)
***Changes in frequency of competition lifts or main multi-joint exercises***	
No change	150 (61.0%)
Decrease	81 (32.9%)
Increase	12 (4.9%)
Other	3 (1.2%)
***Changes in weekly sets***	
Decrease	194 (78.9%)
No change	44 (17.9%)
Increase	8 (3.2%)
Other	0 (0.0%)
***Changes in repetitions per set***	
Decrease	130 (52.8%)
No change	76 (30.9%)
Increase	34 (13.8%)
Other	6 (2.4%)

### Changes in Intensity of Effort During Deloading

Athletes (*n* = 246) reported alterations in the intensity of effort during periods of deloading (Table 6). Athletes stated that there would be a decrease in training intensity for both multi-joint exercises (83.7%) and single-joint exercises (60.2%). Some athletes stated that training intensity would remain unchanged for multi-joint (11.4%) and single-joint (33.3%) exercises. Most athletes reported a decrease in effort and/or proximity to failure for both multi-joint (84.9%) and single-joint (61.8%) exercises.

**Table 6:** Changes in intensity of effort during deloading

	Responses (*n* = 246)
***Changes in intensity (load lifted) for multi-joint exercises***	
Decrease	206 (83.7%)
No change	28 (11.4%)
Increase	8 (3.3%)
Other	4 (1.6%)
***Changes in intensity (load lifted) for single-joint exercises)***	
Decrease	148 (60.2%)
No change	82 (33.3%)
Increase	11 (4.5%)
Other	5 (2.0%)
***Changes in effort/proximity to failure (e.g., RPE/RIR) for working sets for multi-joint exercises***	
Decrease	208 (84.9%)
Increase	20 (8.2%)
No change	15 (6.1%)
Other	2 (0.8%)
***Changes in effort/proximity to failure (e.g., RPE/RIR) for working sets for single-joint exercises***	
Decrease	152 (61.8%)
No change	66 (26.8%)
Increase	21 (8.5%)
Other	7 (2.8%)

### Changes in Exercise Selection and Execution During Deloading

Athletes (*n* = 246) reported how they would adapt exercise selection and execution during deloading. A summary of responses can be found in Table 7. For most athletes, the number of multi-joint exercises would remain unchanged (70.3%) or decrease (26.4%). Similar findings were reported for single-joint exercises, which would also remain consistent (64.2%) or decrease (29.3%) relative to the normal training programme. Athletes reported that the range of motion for exercises performed during the deload would not change. This was reported for both multi-joint (89.0%) and single-joint exercises (90.7%). Where “other” was reported, participants highlighted that “it depends”, but provided no additional information.

**Table 7:** Changes in exercise selection and execution during deloading

	Responses (*n* = 246)
***Changes in number of multi-joint exercises performed?***	
No change	173 (70.3%)
Decrease	65 (26.4%)
Increase	6 (2.4%)
Other	2 (0.8%)
***Changes in number of single-joint exercises performed?***	
No change	158 (64.2%)
Decrease	72 (29.3%)
Increase	15 (6.1%)
Other	1 (0.4%)
***Changes in range of motion for multi-joint exercises***	
No change	219 (89.0%)
Decrease	18 (7.3%)
Increase	7 (2.8%)
Other	2 (0.8%)
***Changes in range of motion for single-joint exercises***	
No change	223 (90.7%)
Decrease	14 (5.7%)
Increase	6 (2.4%)
Other	3 (1.2%)

### Resumption of Training Following Deloading

Athletes (*n* = 246) reported how they would resume their training following a deload. A summary of responses is presented in Table 8. Most athletes states that they would begin a new training block (67.9%), progressively make training hard again (55.3%) or adjust their training based on results from the previous block (50.0%).

**Table 8:** Resumption of training following deloading

	Responses (*n* = 246)
Start a new training block	167 (67.9%)
Progressively make training hard again	136 (55.3%)
Adjust training based on how the previous block went	123 (50.0%)
Start a new programme	73 (29.7%)
‘PR’attempt	18 (7.3%)
Repeat the first week of the previous block	17 (6.9%)
Repeat the week prior to the deload	11 (4.5%)
Repeat the week prior to the deload but with increased volume and/or intensity	11 (4.5%)
No plan, just commencing hard training again	11 (4.5%)

### Assessing the Success of Deloading

Athletes (*n* = 246) reported what they considered to be an effective deload. A summary of responses is in Table 9. Athletes considered a reduction in fatigue (87.8%), increased training motivation (69.5%), and performance improvement (67.5%) to be factors by which a successful deload could be measured. Conversely, an ineffective deload could be the result of continued fatigue (32.5%), a deload that was too short in duration (32.5%), or non-training factors such as life/work circumstances.

**Table 9:** Assessing the success of the deload

	Responses (*n* = 246)
***“How would you know if your deload has been effective?”***	
Fatigue dissipates	216 (87.8%)
Training motivation increases	171 (69.5%)
Performance increases	166 (67.5%)
Aches and pains ease	165 (67.1%)
I don’t know	5 (2.0%)
***“What caused a deload to fail to achieve its purpose?”***	
Fatigue dissipates	216 (87.8%)
Training motivation increases	171 (69.5%)
Performance increases	166 (67.5%)
Aches and pains ease	165 (67.1%)
Trained too heavy	124 (50.4%)
Volume was too high	110 (44.7%)
The deload was too short	80 (32.5%)
Fatigue was too high (e.g., overtraining)	80 (32.5%)
Life/work circumstances	78 (31.7%)
Trained too close to failure	74 (30.1%)
Didn’t adhere to the programmed deload	73 (29.7%)
Injury / illness	69 (28.0%)
I don’t know	5 (2.0%)

### Deloading Enjoyment

Athletes (*n* = 246) reported what they do/do not enjoy about deloading. A summary of responses can be found in Table 10. Some athletes (43.1%) reported that they had neutral feelings about deloading, with others reporting that they either enjoy deloads (39.4%) or do not enjoy them (15.9%). “Other” responses related to athletes “sometimes” enjoying deloads, with one athlete reporting that “I really hate them and only do them because I know it aids performance, but it makes me feel lazy”. The main reasons for enjoying deloading included increased recovery (27.2%), reduced psychological burden (25.2%), and that training sessions were easier (22.8%). Those that did not enjoy deloading stated that they disliked not training hard (12.6%).

**Table 10:** Deloading enjoyment

	Responses (*n* = 246)
***“Do you enjoy deloads?”***	
Neutral	106 (43.1%)
Yes	97 (39.4%)
No	39 (15.9%)
Other	4 (1.6%)
***“What do you enjoy about deloading?”***	
Increased recovery	67 (27.2%)
Less psychological burden	62 (25.2%)
Easier training sessions	56 (22.8%)
More time available for other activities	52 (21.1%)
Decrease in soreness	43 (17.5%)
More energy	43 (17.5%)
Improved injury management	32 (13.0%)
***What do you not enjoy about deloading?***	
Not training hard	31 (12.6%)
Less time spent training	16 (6.5%)
Losing ‘touch’ with the main lifts	10 (4.1%)
Lack of ‘pump’	9 (3.7%)
Lack of soreness	4 (1.6%)
Disrupted lifestyle/schedule	4 (1.6%)
Looking worse	3 (1.2%)

### Recovery Modalities

Athletes (*n* = 246) were asked what recovery modalities they employ in conjunction with deloading. A summary of responses is presented in Table 11. There were 118 (48.0%) athletes that utilised concurrent recovery strategies during periods of deloading. The most reported were massage (28.9%), static stretching (25.6%), and foam rolling (24.8%).

**Table 11:** Recovery modalities

	Responses (*n* = 246)
***“Do you use additional recovery modalities?”***	
No	128 (52.0%)
Yes	118 (48.0%)
***“What recovery strategies do you use in conjunction with deloads?”***	
Massage	71 (28.9%)
Static stretching	63 (25.6%)
Foam rolling	61 (24.8%)
Heat exposure	28 (11.4%)
Nutritional changes	27 (11.0%)
Cold exposure	17 (6.9%)
Dry needling	11 (4.5%)
Taping	4 (1.6%)

### Deloading Education

Athletes (*n* = 246) reported how they educate themselves on deloading. A summary of responses is presented in Table 12. The most common education method reported by athletes was published literature (69.9%) followed by past experience (68.3%) and the athlete’s coach (59.3%).

**Table 12:** Deloading education

	Responses (*n* = 246)
Literature	172 (69.9%)
Past experience	168 (68.3%)
Coach	146 (59.3%)
Other athletes	86 (35.0%)

## Discussion

At the time of writing, research investigating deloading within strength and physique sports was notably absent within the literature, despite a growing body of evidence exploring tapering practices [20–23]. To our knowledge, this study is, therefore, the first to document the deloading practices of competitive strength and physique athletes and explored both reasons provided for deloading, experiences of deloading, and approaches taken to do it. We benefited from respondents who participated in a range of strength and physique sports and were able to gain insight into the practices of individuals across various training and competitive characteristics, including those competing internationally, and those who were coached and self-coached. The information in this study will therefore assist athletes, coaches, and sports scientists in better understanding how deloading is undertaken in practice across a range of groups that participate in strength and physique sports and provides a helpful foundation for further work to expand upon our preliminary findings through further empirical investigation.

The typical duration of a deload reported by athletes here was 6.4 ± 1.8 days, integrated into training every 5.8 ± 3.4 weeks to preserve energy and manage fatigue through a reduction in total training stress. Restorative microcycles such as these feature within periodisation systems that strategically manipulate adaptive training responses to facilitate the long-term, progressive development of physical performance, whilst ameliorating the negative (‘fatigue’) effects of hard exercise [15]. Similarly, tapers typically last for ~ 7 days following periods of higher training stress, to promote restoration following phases of high training demand and to optimise competition readiness [20–23, 26]. Whilst they fundamentally serve similar purposes, deloading aims to facilitate progression within day-to-day training as part of a longer-term plan whereas tapering can be thought of as an acute strategy to elicit peak competition performance through the management and mitigation of training adaptations and fatigue-related responses [19]. Deloading was undertaken proactively through pre-programmed reductions in training stress for a proportion of respondents; a combination of pre-planned and reactive deloading strategies was also relatively typical; relying solely on reactive deloading using an autoregulated strategy for some – that is, flexibly, based primarily on biofeedback gained during training – was much less common, however. It was interesting to note that deloading through a combination of reactive and pre-planned means was relatively normal amongst respondents, but that solely relying on reactive strategies was comparatively unusual.

Autoregulated approaches to programming now feature throughout the literature, where training is adjusted flexibly based on individual rates of adaptation using tools, methods, and techniques such as Autoregulatory Progressive Resistance Exercise (APRE), Ratings of Perceived Exertion (RPE), Repetitions in Reserve (RIR) and Velocity-Based Training (VBT) [31], amongst others. Whilst growing data points to the utility of methods such as these [32], research exploring the use of reactive/autoregulatory deloading strategies is markedly wanting, however. Emerging insight elucidates interindividual responses to resistance training, where trainees adapt at different rates and magnitudes for a given stimulus [33, 34]. Along with the recognition that lifestyle, phenotype, and psychosocial factors external to training can affect individuals’ recovery and adaptive potential [28], this variability underpins autoregulated training [31]. Indeed, recent data highlights that coaches perceive that factors outside of physical training can heavily influence an athlete’s adaptive response to that training, including life, psychological and emotional stresses [35]. This means that life-related factors outside of pre-programmed training might influence an individual’s ability to adapt to it appropriately. Indeed, psychological stressors (such as life events or perceived stresses) appear to impair recovery and motor function following resistance training [36, 37]. It stands to reason therefore that managing recovery through reactive strategies that quickly respond to harmful responses (such as training ‘fatigue’ and/or stress) in an individualised manner is also prudent and extends the existing autoregulation inquiry into the realms of regeneration as well as training. Future research should therefore explore autoregulated methods (including combined reactive and pre-programed methods) to systematically *reduce* training stress within periodised strength and physique training programmes, to facilitate long-term progression, alongside those that aim to improve performance.

Athletes here stated that deloading was undertaken to preserve energy, manage/dissipate ‘fatigue,’ and prepare for the next block of training. Most athletes suggested that they felt that they could continue to progress their training without deloading, indicating that they felt that it might not be a necessity day to day. Similarly, our earlier study with the coaches of national and international level strength and physique athletes also revealed that some felt that deloading might not be a prerequisite for progressive training too [26]. Conceivably, this could highlight a degree of inter-athlete variability in the requirement for deloading, or that some coaches and athletes might value deloading more/less than others within training. Interestingly, a recent article highlighted that a one-week period of no training at the midpoint of a 9-week resistance training programme negatively impacted lower body strength – but not hypertrophy, power or local muscular endurance – when compared with continuous training [38]. This suggests that the complete cessation of training might be detrimental for maximal strength where neural adaptations and exposure to load are an important antecedent to performance [39]. The periodic absence of training within a short-to-medium term programme might therefore be detrimental to some trainees, and more work might need to be done to understand optimal deloading strategies to manage training load for purposes such as maximal strength training, hypertrophy, and rapid force production – a ‘one-size fits all approach’ would appear to conflict with the emerging experiential and empirical evidence.

The athletes here also reported mixed perspectives around their experience of deloading; some explained that they enjoyed the increased recovery that deloading provides as well as the reduced physical and psychological burden of not needing to push training constantly; others were neutral, and some did not enjoy deloading nor the reduced training of the deloading phase. For some, this highlighted that deloading might provide important psychological relief as well as physical benefits; for others, deloading might be perceived as an annoyance. Surprisingly, whilst data points to psychological benefits from engaging in resistance exercise [40, 41], there is a scarcity of data exploring the physical and psychological burden of progressive strength training. To explain, resistance training acts as an acute stressor that leads to physiological responses within the sympathetic and parasympathetic nervous system that antedate psychological stress [42]. It has been argued that training in general should be viewed as a biopsychosocial process with complex physical and psychological interactions [43], and recall that recent insight from coaches highlights that stress responses are perceived to markedly affect athletes’ response to training [35]. Allostatic load is the cumulative effect of chronic exposure to the perceived environmental and physical stressors [45], and while exposure to stress is an important antecedent to adaptation [45], repeated stress without sufficient recovery is harmful [46]. The physical requirement to undertake strength training, coupled with the psychological burden of difficult, progressive training – and, in the case of weight-making and aesthetic sports (such as physique sports), the periodic need for negative energy balance too – might lead to accumulated allostatic loading and negative health consequences manifesting over time if not carefully managed [44, 46]. Indeed, training for sport can be an exhaustive experience punctuated by psychological requirements to perform day-to-day and within competition [47]. Phasic periods to reduce allostatic loading (perhaps through reduced training stress) might be required for longer-term wellbeing as well as progression.

Most athletes deloaded by reducing overall training volume and reducing sets and repetitions whilst maintaining frequency and exercise selection, by reducing intensity by lifting less when completing single and multi-joint exercises, and by increasing repetitions in reserve (defined as the number of repetitions a trainee perceives that they complete within a set prior to reaching muscular failure, [48]). Deloading was therefore achieved by reducing intensity of effort as well as through the manipulation of the traditional metrics of absolute volume and intensity. Interestingly, the frequency within which some respondents chose to deload varied markedly, with some appearing to deload biweekly, perhaps undulating between challenging and easier microcycles, and with others appearing to deload after an extended period. Similarly, the length of the deload also varied, with some deloading for only one day whilst others deloaded for much longer. Indeed, the variability in practices reported here could reflect the interindividual responses highlighted previously [33, 34], which might necessitate individualised approaches to restoration, or different approaches taken to periodise training [15, 17, 43] leading to different strategies taken to deload.

Tapering recommendations and practices (for strength athletes) that appear within the literature include reducing volume by ~ 30–70% whilst maintaining intensity ≥ 85% 1RM, using an exponential or step-like taper for 1–2 weeks prior to competition, and with a short cessation period of 2–7 days [19–23]. Whilst respondents here reduced overall training volume during their deloads in a similar manner to that reporting in the tapering literature, the maintenance of intensity appears to be a defining factor that differentiates the two strategies [19–23]. For the most part, participants here reported an overall reduction in intensity during the deload, whereas data from the tapering literature highlights that intensity is mostly maintained (or increased in some cases) alongside the reduction in volume [19–23]. Indeed, training specificity typically increases in the weeks leading up to competition [45], and within strength sports intensity is an important programming variable for the achievement of specificity considering that the purpose of these activities is to lift the maximum load within the parameters of the activity’s ruleset. Fundamentally, tapering is a tool to facilitate competition readiness (where intensity is important in the build-up); deloading is a tool to facilitate day-to-day training and facilitate longer-term progress.

Whilst research exploring tapering and peaking strategies is relatively well established and provides some degree of consensus [19, 20], similar data for deloading is comparatively absent, however. Conceivably, approaches taken to tapering could be adapted and followed for deloading and might offer some degree of stability if this is familiar to the athlete and that responses are reliable. Recall that we noted that a small proportion of respondents here disliked deloading, despite most agreeing that outcomes of a successful deload appear to be beneficial, presumably due to high levels of intrinsic motivation to train. If responses to deloading are reliable (as in, through a rebound in performance and improvement in wellbeing) and somewhat analogous to tapering—where athletes might have experienced success previously—this might improve athletes’ acceptance and perceptions of deloading, and motivations to do so within training appropriately.

Following the deloading period, most respondents would begin a new phase of training; training would be made progressively more challenging; and training would be adjusted based on the previous block. A combination of factors would enable participants to judge whether the deload was effective, including the dissipation of fatigue, increases in motivation and performance, and reductions in aches and pains; training too heavy and with too much volume were revealed to be reasons for unsuccessful deloads. Interestingly, emerging evidence highlights that molecular responses that mediate adaptations become blunted to repeated exposure from training, meaning that anabolic signalling as a response to strength training might reduce over time [49–51]. This could partially explain stagnation and accommodation to training [45] and provides a physiological rationale for periodically deloading. Indeed, it appears that periodic unloading re-sensitises signalling [49, 50], meaning that this is a potential means to facilitate longer-term adaptation as well as manage fatigue. Whilst some evidence has utilised periods of no training to elicit re-sensitisation [49], it also appears that ‘active’ recovery periods of reduced volume and intensity through a deload – whilst maintaining frequency and exercise selection like the approaches athletes undertook here – also leads to similar effects without the need to cease training completely, for a short period [52]. This is important, as motivated athletes (such as some of those who responded here) might be more likely to reduce training through a deload than to eliminate it completely. Data exploring the longer-term benefits of periodic unloading within the context of progressive training is absent at the time of writing however, and more work needs to be done to investigate if deloading facilitates greater strength and hypertrophy improvements than continuous exercise over time. For the time being, the emerging data provides only preliminary evidence for potential mechanisms of action and possible benefits if implemented periodically.

Nearly half of the respondents reported that they supplemented their training with ‘recovery’ modalities such as massage (28.9%), foam rolling (24.8%) and static stretching (25.6%). A wide variety of approaches to addressing recovery have featured throughout the literature, including “active” recovery methods utilising submaximal activity to expedite a shift from stress-induced physiologic disturbance towards physiologic stability through restorative movement [52], and “passive” methods including massage techniques, cryotherapy and compression garments and devices that aim to facilitate regeneration through external stimulation [53]. Recently, “proactive” recovery strategies such as breathing techniques and other self-initiated methods have also been discussed [54] and have gained popular interest outside of the literature. Interestingly, data supporting the use of recovery techniques appears to be ambiguous however, with literature suggesting that techniques such as massage [55], cryotherapy and compressions garments might offer some benefit [56], that stretching [57] and foam rolling [53, 58]  might not, and that active recovery strategies might offer some psychological advantage despite physiologic and performance benefits not being certain [52]. What is clear is that recovery is a complex, multifaceted psychobiological process and that no unitary marker exists that adequately encompasses its aetiologic multiplicity [54, 55]. Indeed, future work might need to explore similarly complex recovery interventions that purposely attend to performance, physiological, and perceptual recovery markers either in combination or sequence relevant to their time course of decay.

Limitations of this research include unequal group sizes for the respondents; most athletes here participated in Powerlifting, meaning that representation from other sports such as Weightlifting and Strongman/woman was less and that the findings might be most applicable to individuals of that sport specifically. Similarly, a large proportion of respondents here were coached athletes (45.9%), meaning that this subsample did not actively programme their own deload strategies and were not the decision-makers in their day-to-day training. That said, we were able to recruit participants who were competitive at the international and national level across the globe, from both sexes, across multiple sports, and provide unique insight into an important – and under investigated – aspect of training. Similarly, whilst our survey offered some insight into participants’ experiences of deloading, its design was limited to multiple-choice responses, meaning that qualitative data was not collected. Future work could expand upon some of our findings and provide the in-depth exploration of perceptions and experiences around fatigue and deloading within strength and physique sports through qualitative means and would offer rich insight into the lived experiences of these aspects of athletes’ training. This might help to understand some of the psychological aspects of training and deloading that were introduced here and begin to address some of the emerging criticisms made of the literature, which might have emphasised biological responses without attending sufficiently to psycho-emotional experience of training [43, 44]. Finally, this study did not formerly observe the Checklist for Reporting Survey Studies (CROSS) [59], and so whilst we made every attempt to adhere to good practice principles, some aspects of the CROSS tool such as respondents’ country of origin have not been provided (part of the sample characteristics criterion). Indeed, we were unable to locate respondents’ geolocation data, and were therefore unable to report respondents’ geographical information. Readers of this article will need to be mindful that we were unable to provide this data, which remains a limitation in the reporting of this study.

## Conclusions

Findings from this study highlight common deloading characteristics and methods of competitive strength and physique athletes that may assist others in conceptualising, designing, and implementing deloading into their training programmes or research. These characteristics include a general reduction in training volume and intensity of effort that is approached using autoregulation and pre-programmed strategies undertaken when the athlete experiences unexpected fatigue or muscle soreness or to pre-empt its manifestation. It is worth noting that there is a clear lack of empirical research exploring the utility of deloading in strength and physique sports and therefore the findings from this study act only as broad guidelines for the development of deload training until further experimental research using robust methodologies elucidates its value in a practical training environment. Until this point, any recommendations are based on triangulating anecdotal practices along with evidence elsewhere.

## Data Availability

The datasets generated during and/or analysed during the current study are available in the Open Science Framework repository [https://osf.io/jmkpg/?view_only=354ffdd17fab4b3086563c9c6e8199f4].

## Declarations

### Author’s Information

N/A.

### Rights and Permissions

For the purpose of open access, the author has applied a Creative Commons Attribution (CC BY) licence to any Author Accepted Manuscript version arising from this submission.

## Abbreviations

APRE: Autoregulatory Progressive Resistance Exercise
RPE: Ratings of Perceived Exertion
RIR: Repetitions in Reserve
VBT: Velocity-Based Training
